# Biocontrol Potential of Endophytic *Streptomyces malaysiensis* 8ZJF-21 From Medicinal Plant Against Banana Fusarium Wilt Caused by *Fusarium oxysporum* f. sp. *cubense* Tropical Race 4

**DOI:** 10.3389/fpls.2022.874819

**Published:** 2022-05-11

**Authors:** Lu Zhang, Ziyu Liu, Yong Wang, Jiaqi Zhang, Shujie Wan, Yating Huang, Tianyan Yun, Jianghui Xie, Wei Wang

**Affiliations:** ^1^Ministry of Education Key Laboratory for Ecology of Tropical Islands, Key Laboratory of Tropical Animal and Plant Ecology of Hainan Province, College of Life Sciences, Hainan Normal University, Haikou, China; ^2^Key Laboratory of Biology and Genetic Resources of Tropical Crops, Institute of Tropical Bioscience and Biotechnology, Chinese Academy of Tropical Agricultural Sciences, Ministry of Agriculture, Haikou, China; ^3^Haikou Experimental Station, Chinese Academy of Tropical Agricultural Sciences, Haikou, China

**Keywords:** endophytic *Streptomyces*, biocontrol, banana Fusarium wilt, genome sequencing, antifungal mechanism

## Abstract

Banana (*Musa* spp.) is an important fruit crop cultivated in most tropical countries. Banana Fusarium wilt caused by *Fusarium oxysporum* f. sp. *cubense* tropical race 4 (*Foc* TR4) is the most destructive fungal disease. Biocontrol using endophytic microorganisms is considered as a safety and sustainable strategy. Actinomycetes have a potential for the production of diverse metabolites. Isolation of endophytic actinomycetes with high efficiency and broad-spectrum antagonism is key for exploring biocontrol agents. Our previous study showed that a total of 144 endophytic actinomycetes were isolated from different tissues of medicinal plants in Hainan, China. Especially, strain 8ZJF-21 exhibited a broad-spectrum antifungal activity. Its morphological, physiological, and biochemical characteristics were consistent with the genus *Streptomyces.* The phylogenetic tree demonstrated that strain 8ZJF-21 formed a distinct clade with *Streptomyces malaysiensis*. Average nucleotide identity (ANI) was 98.49% above the threshold of novel species. The pot experiment revealed that endophytic *Streptomyces malaysiensis* 8ZJF-21 could improve the plant resistance to *Foc* TR4 by enhancing the expression levels of defense-related and antioxidant enzyme genes. It also promoted the plant growth by producing several extracellular enzymes and metabolites. Antifungal mechanism assays showed that *S. malaysiensis* 8ZJF-21 extract inhibited mycelial growth and spore germination of *Foc* TR4 *in vitro.* Pathogenic cells occurred cytoplasmic heterogeneity, disappeared organelles, and ruptured ultrastructure. Sequencing and annotation of genome suggested that *S. malaysiensis* 8ZJF-21 had a potential of producing novel metabolites. Nineteen volatile organic compounds were obtained from the extract by Gas Chromatography-Mass Spectrometry (GC-MS). Hence, endophytic *Streptomyces* strains will become essential biocontrol agents of modern agricultural practice.

## Introduction

Bananas (including plantains and other cooking bananas) are the world’s most important fruit with a global production of 113.9 million tons (Nansamba et al., 2020). They are also a staple food for millions of people throughout the developing world (Kema et al., 2020). In Africa, over 70 million people derive 25% of their dietary energy from bananas and plantains (Nansamba et al., 2020). Vegetative propagation of commercial cultivars results in a narrow genetic background, making plants susceptible to various pathogens, especially banana Fusarium wilt caused by *Fusarium oxysporum* f. sp. *cubense* (*Foc*) (Wang et al., 2012). The pathogen contains at least four races based on the pathogenicity to host cultivars. *Foc* tropical race 4 (*Foc* TR4) is the most destructive fungal disease. It can infect Cavendish banana and all cultivars that are sensitive to the other three races (Ploetz, 2015). Chemical control using fungicides is minimally effective (Jing et al., 2020; Wei et al., 2020). The intensive use results in the pathogenic resistance to fungicides and an increase in environmental contamination (Dita et al., 2018). No commercial varieties display an effective resistance against *Foc* TR4 until now (Dale et al., 2017; Wang et al., 2021). The presence of any resistant cultivars would not exclude the use of other disease control approaches that could contribute to maintaining the resistance to pathogens over time. Biological control is an eco-friendly strategy to manage soil-borne phytopathogens. Disease-suppressive soil provides the best example of microbe-associated defense against the invasion of *Foc* TR4 (Zhou et al., 2019). Therefore, the establishment of an effective way to stimulate the accumulation of beneficial microorganisms and decrease the abundances of pathogenic *Fusarium* is critical for the successful management of banana Fusarium wilt.

Beneficial microorganisms are an important source of agricultural biocontrol agents. Recently, endophytes have received considerable attention for their potential to control fungal phytopathogens (De Silva et al., 2019). They colonize mainly the root system and the xylem tissues of host plants, developing a mutualistic relationship to induce plant defense response toward various pathogens and promote plant growth. Among these endophytic microorganisms, the phylum Actinobacteria are reported as an important portion (Palaniyandi et al., 2013; Dinesh et al., 2017). Most endophytic actinomycetes isolated to date mainly belong to the genus *Streptomyces* (Golinska et al., 2015; Vurukonda et al., 2018). Previous studies reported the role of *Streptomyces* in the biocontrol of soil-borne phytopathogens such as *Foc* TR4 (Yun et al., 2021), *Glomerella cingulata* (Marian et al., 2020), *Sclerotium rolfsii* (Singh and Gaur, 2016), *Botrytis cinerea* (El-Shatoury et al., 2020), and *Alternaria brassicicola* (Hassan et al., 2017). The success of *Streptomyces* as a potential biocontrol agent encourages research into new microbial agents as alternatives to chemical fungicides (Dhanasekaran et al., 2005; Jing et al., 2020; Zhang et al., 2021).

Indeed, the antagonistic activity of *Streptomyces* spp. against phytopathogens is related to the production of antimicrobial compounds including antibiotics, enzymes, and alkaloids (Lacey and Rutledge, 2022). Among approximately 23,000 of the identified bioactive metabolites produced by microorganisms, about 7,600 compounds were found from the genus *Streptomyces* (Olanrewaju and Babalola, 2019). About 80% of the bioactive compounds for agricultural and medical use originate from the genus *Streptomyces* (Ferraiuolo et al., 2021). To discover novel biocontrol candidates, some researchers attempted to isolate endophytic actinomycetes from various medicinal plants (Passari et al., 2015; Ayswaria et al., 2020; Li et al., 2020; Musa et al., 2020; Yun et al., 2021). For example, 12 out of 68 endophytic actinomycetes isolated from six medicinal plants reduced the infection of collar rot caused by *Sclerotium rolfsii* in chickpea (Singh and Gaur, 2016). Five endophytic *Streptomyces* in the traditional medicinal plant *Arnica montana* produced a huge variety of bioactive secondary metabolites (Wardecki et al., 2015). Twenty-two endophytic actinomycetes recovered from medicinal plants exhibited inhibitory activity against at least one pathogen (Passari et al., 2015). Recent study showed that precious bioactive compounds produced by medicinal plants contribute to the natural regeneration of endophytes to cope with stressful conditions (Wu et al., 2021). The genomic evolution is beneficial for endophytes to produce novel bioactive compounds. Thus, endophytic *Streptomyces* from medicinal plants may have great potential as biocontrol agents.

In our previous study, 144 endophytic actinomycetes were isolated from different tissues of 23 medicinal plants. The antagonistic experiment showed that strain 8ZJF-21 had strong antifungal activity against *Foc* TR4. Here, our study’s aim was to investigate the properties of the endophytic strain 8ZJF-2 from the roots of *Curculigo capitulata*. We first identified the species and genus of strain 8ZJF-2 and determined its broad-spectrum antifungal activity *in vitro*. Biocontrol efficiency and antifungal mechanism against *Foc* TR4 were further evaluated. To assay the potential ability to produce the antifungal metabolites, genomic sequencing and Gas Chromatography-Mass Spectrometry (GC-MS) were performed. Our results will provide a promising endophyte for controlling banana Fusarium wilt.

## Materials and Methods

### Antifungal Bioassay of Endophytic Actinomycete Strain 8ZJF-2 Against *Foc* TR4

A total of 144 endophytic actinomycetes were isolated previously from different tissues of 23 medicinal plants in “Wuzhishan” Nature Reserve, Hainan, China the related data will be published in Phytopathology, but the publication period is a little long. Antagonistic activity was evaluated against *Foc* TR4 (ATCC 76255) *in vitro* as previously described (Jing et al., 2020). *Foc* TR4 was cultured on the potato dextrose agar (PDA) medium at 28°C for 7 days. An agar disc (5 mm in diameter) with *Foc* TR4 was placed on the center of Petri dishes 6 cm away from an endophytic actinomycete. Plates without endophytic actinomycetes were served as a control. After inoculation at 28°C for 10 days, the inhibition percentage was calculated as described by Wei et al. (2020). All experiments were performed in triplicate. The endophytic strain 8ZJF-2 isolated from the roots of *C. capitulata* exhibited strong antifungal activity.

### Assaying a Broad-Spectrum Antifungal Activity of Strain 8ZJF-2

To further analyze whether strain 8ZJF-2 owned a broad-spectrum antifungal activity, the antagonistic activities were investigated against ten phytopathogenic fungi, including *Curvularia lunata* (ATCC 42011) from banana, *Colletotrichum fragariae* (ATCC 58718) from strawberry, *Fusarium oxysporum* f. sp. *cucumerinum* (ATCC 36332) from cucumber, *Fusarium graminearum* Sehw (ATCC 11696) from wheat, *Fusarium oxysporum* f. sp. *cubense* race 1 (ACCC 31271) from banana, *Colletotrichum gloeosporioides* (Penz) Penz and Sacc 1884 (ACCC 36351) from mango, *Pyricularia oryzae* (ATCC 52083) from rice, *Alternaria tenuissima* (ATCC 58124) from cotton, *Colletotrichum acutatum* (ATCC 56815) from loquat, and *Colletotrichum gloeosporioides* (ATCC 16330) from mango. Antifungal activity of strain 8ZJF-2 was detected as the above-mentioned method. The inhibition zones were measured in millimeters (Zhang et al., 2021).

### Morphological, Physiological, and Biochemical Characteristics of Strain 8ZJF-2

The strain 8ZJF-21 was inoculated in various types of growth media including PDA and different International *Streptomyces* Project media (ISP2, ISP3, ISP4, ISP5, ISP6, and ISP7) for 7 days at 28°C under dark conditions (Yun et al., 2021; Zhang et al., 2021). Cultural characteristics such as colonial morphology and diffusible pigment production were detected in the different media according to Shirling and Gottlieb (1966). Based on Bergey’s manual of systematic bacteriology, strain 8ZJF-21 was classified by observing the color of aerial and substrate mycelia (Brinley-Morgan and McCullough, 1974). Phenotypic profile of strain 8ZJF-2 spore chain was observed by scanning electron microscopy (SEM, model S-4800, Hitachi Limited, Japan). Utilization of nitrogen and carbon sources was studied according to Qi et al. (2019). The capability of strain 8ZJF-21 to produce important enzymes (proteases, lipases, celluloses, nitrate reductases, pectinases, gelatinases, and ureases), indoleacetic acid (IAA), siderophores, and H_2_S were determined (Jing et al., 2020; Wei et al., 2020; Zhang et al., 2021). Physiological tests were performed by inoculating strain 8ZJF-21 on the selected medium (ISP2) at different temperatures (20°C–50°C), pH (3.0–11.0), and NaCl (0–20% w/v).

### Genomic Sequencing and Functional Annotation of Strain 8ZJF-21

Strain 8ZJF-21 was cultured in the ISP2 liquid medium at 200 rpm and 28°C for 4 days. Total genomic DNA was extracted using a Rapid Bacterial Genomic DNA Isolation Kit (Biotake corporation, Beijing, China). The sequencing libraries were generated using the Illumina TruSeq™ RNA Sample Preparation Kit (Illumina, San Diego, CA, United States). The complete genome was sequenced in the Illumina Hiseq × Ten platform (Illumina, San Diego, CA, United States) by the Shanghai Majorbio Bio-pharm Technology Co. Ltd. Sequencing data were analyzed using an online platform of the Majorbio Cloud^1^ and was deposited in GenBank with accession number JAJQWY000000000. The open reading frames (ORFs) were predicted by the Rapid Annotation using Subsystem Technology (Brettin et al., 2015). Functional annotation was performed using the Clusters of Orthologous Group (COG), the Gene Ontology (GO), and the Kyoto Encyclopedia of Genes and Genomes (KEGG) (Ogata et al., 1999; Tatusov et al., 2000). Biosynthetic gene clusters (BGCs) were identified by the online antiSMASH v4.0.2 software (Weber et al., 2015).

### Construction of Phylogenetic Trees

The 16S rDNA sequence was extracted from the sequenced genome of strain 8ZJF-21. Sequence alignment was performed against the EzTaxon-e database.^2^ The phylogenetic trees were constructed using a neighbor-joining (NJ) method of MEGA 7.0 (Kumar et al., 2016). Evolutionary distance was calculated using the maximum-parsimony algorithm. The confidence level was calculated using the bootstrap analysis on 1,000 replicates. Average nucleotide identity (ANI) was obtained by comparing genomes of the type strain and strain 8ZJF-21 using the online OrthoANI (Yoon et al., 2017). The closest homolog was considered as the type strain according to the phylogenetic tree. Its genome sequence was downloaded from the database of EzBioCloud.^3^

### Extraction of Strain 8ZJF-21 Metabolites

Strain 8ZJF-21 was cultured in 100 ml of a soybean liquid medium (SLM, 15 g of corn flour, 10 g of glucose, 0.5 g of K_2_HPO_4_, 0.5 g of NaCl, 0.5 g of MgSO_4_, 3 g of beef extract, 10 g of yeast extract, 10 g of soluble starch, 2g of CaCO_3_, pH 7.2-7.4) with shaking at 180 rpm for 7 days at 28°C. The fermentation broth was filtered through a Whatman No.1 filter. After centrifugation at 10,000 rpm for 15 min, the supernatant was extracted twice in the ratio of 1:1 (culture supernatant: different gradient methanol). To remove the impurities, the suspension went through a silica-gel chromatography column (5.5 cm × 80 cm, inner diameter × length). The elution with gradient methanol solutions was filtered through a 0.22 μm sterile filter (Millipore, Bedford, MA, United States) (Li et al., 2021). The organic solvent was concentrated using a rotary vacuum evaporator (N-1300, EYELA, Ailang Instrument Co., Ltd., Shanghai, China). The obtained extract was redissolved in 10% (v/v) of dimethyl sulfoxide (DMSO) with a final concentration of 20 mg ml^–1^.

### Antifungal Activity of Extract Against *Foc* TR4

Sterilized PDA agar media containing final extract concentrations (1.563, 3.125, 6.25, 12.50, 25, 50, or 100 mg L^–1^) were prepared by a serial dilution method (Yun et al., 2021). Ten percent (v/v) of DMSO was used as a control. A 5-mm-diameter disc of *Foc* TR4 was placed on the center of the plate. The growth diameter of *Foc* TR4 was recorded until the mycelia reaching the plate edge in the control group. The half-maximal effective concentration (EC_50_) of the extract against *Foc* TR4 was calculated according to Hoekstra and Van Ewijk (1993). All experiments were repeated with three biological replicates.

### Biocontrol Evaluation and Plant-Growth Promoting of Strain 8ZJF-21

To investigate the potentiality of strain 8ZJF-21 to control *Foc* TR4 and promote plant-growth traits, a pot experiment was carried out in a completely randomized design. *Foc* TR4-GFP strain overexpressing a green fluorescent protein (GFP) gene was selected to detect the infection in banana roots (Yun et al., 2021). *Foc* TR4-GFP was prepared by incubation in PDB (potato dextrose broth) in a rotary shaker (180 rpm) for 5 days at 28°C. The liquid culture was then filtered through four layers of sterile gauze. The spores were enumerated by hemocytometer under a light microscope (Axio Scope A1, Carl ZEISS, Germany) and were then diluted to 1.0 × 10^5^ cfu/mL with sterile water. Strain 8ZJF-21 was inoculated in one liter of an Erlenmeyer flask containing 300 ml of sterilized SLM at 200 rpm and 28°C for 7 days. The suspension was diluted to the final concentration of 1 × 10^5^ cfu/ml. Spore suspension of *Foc* TR4-GFP (100 ml) and strain 8ZJF-21 (100 ml) was completely mixed with 10 g of autoclaved soil. The banana seedlings (*Musa* AAA group, Cavendish cv. Brazil) with four to five leaves were transferred to the pots (12 cm in diameter). These banana seedlings were kept in a glasshouse under natural light at 28°C ± 2°C. Three experiment groups were set including sterilized SLM + *Foc* TR4-GFP (1 × 10^6^ spores/g soil) (G1), fermentation broth of strain 8ZJF-21 (1 × 10^6^ spores/g soil) + *Foc* TR4-GFP (1 × 10^6^ spores/g soil) (G2), and sterilized SLM (G3). All experiments were performed in triplicates. Each group contained 60 pots with three replicates. After 0.5, 1, 2, 3, 4, and 5 days post inoculation (dpi), the root samples of banana seedlings were collected for determining the expression levels of defense-related and antioxidant enzyme genes. The chlorotic symptom of banana leaves was monitored at 30 dpi. The disease indexes were recorded according to Li et al. (2021). *Foc* TR4-GFP infection in banana roots was detected by a confocal microscope (FV1000-IX81, Olympus, Japan). The physiological parameters of banana seedlings were measured at 30 dpi, including stem diameter, chlorophyll content, leaf area, dry weight, fresh weight, plant height, and leaf thickness (Zhang et al., 2021).

### Measurement of H_2_O_2_ and Malondialdehyde in Roots of Banana Seedlings

As above mentioned, roots treated with strain 8ZJF-21 and/or *Foc* TR4 were collected at 0.5, 1, 2, 3, 4, and 5 dpi. H_2_O_2_ was measured according to Ferguson et al. (1983). One gram of frozen sample was ground in 5 ml of pre-cooled acetone. After centrifugation at 10,000 rpm for 20 min at 4°C, the supernatant was mixed with 0.5 ml of TiCl_4_ (20% v/v TiCl_4_ in concentrated HCl). And then, 3.5 ml of NH_4_OH was added dropwise with thorough mixing. Following centrifugation, the precipitates were redissolved in 25 ml of H_2_SO_4_ (2 mol/L). The absorbance was recorded at 415 nm. A blank without the addition of sample was made through the same procedure. The standards ranging from 0.15 to 0.75 mol L^–1^ H_2_O_2_ were also reacted with TiC14. Malondialdehyde (MDA) was determined using the reaction method of thiobarbituric acid. Four grams of frozen samples were ground in 15 ml of trichloroacetic acid (5%, w/v). After centrifugation at 6,000 rpm for 10 min, 1.5 ml of the supernatant were mixed with 2.5 ml of thiobarbituric acid (0.5%, w/v) in 15% of trichloroacetic acid. The mixture was incubated at 100°C for 20 min. Absorbance of the supernatant was recorded at 532 nm and corrected using non-specific turbidity by subtracting the absorbance at 600 nm. The content of MDA was calculated according to Pongprasert et al. (2011) and expressed as mol g^–1^ FW.

### Expression Analysis of Defense-Related Genes by Quantitative Real-Time Polymerase Chain Reaction

The total RNA of banana roots was extracted using the method of Trizol (Wang et al., 2014). The quality and quantity of RNA were measured by Nanodrop (Thermo Scientific, United States). The first-strand cDNA was synthesized using the Prime Script*™* RT Reagent Kit with gDNA Eraser (Takara, Dalian, China). Quantitative real-time polymerase chain reaction (qRT-PCR) was performed in a LightCycler^®^ 480 System (Roche Diagnostics, Mannheim, Germany) with the SYBR Premix Ex Taq II kit (Takara, Dalian, Liaoning, China). Four defense-related marker genes such as β-1,3-glucanase (*Ma*β*-1,3-Glu*, GenBank ID: AF001523), mitogen-activated protein kinase 1 (*MaMAPK1*, GenBank ID: XM018826311), phenylalanine ammonia lyase (*MaPAL*, GenBank ID: XM009403673), and pathogen-related protein 1 (*MaPR-1*, GenBank ID: XM009388962) were selected. The primer sequences were listed in Supplementary Table 1. The reaction system of qRT-PCR was described in our previous study (Zhang et al., 2019). The house-keeping gene of 18S *rRNA* (GenBank ID: U42083) was used as a reference gene to normalize the expression levels of target genes using the 2^–ΔΔCt^ method (Wang et al., 2014). All experiments were repeated in triplicates with at least three biological replicates of each sample.

### Effect of Extract on Spore Germination of *Foc* TR4

*Foc* TR4 was cultured in the potato dextrose broth (PBD) at 200 rpm for 7 days at 28°C. After filtration through six layers of gauze to remove hyphae, spores were collected at 1,000 rpm for 10 min and washed using sterile water four times. The spore suspension (1 × 10^6^ spores/mL) of *Foc* TR4 was prepared using sterile water (Wang et al., 2012). The effect of the extract on spore germination of *Foc* TR4 was evaluated according to Wei et al. (2020). Briefly, different concentration extracts (1 × EC_50_, 2 × EC_50_, 4 × EC_50_ and 8 × EC_50_) of strain 8ZJF-21 and *Foc* TR4 (1 × 10^6^ spores/mL) were mixed completely and added to the concavity of slide. *Foc* TR4 spores treated with 10% (v/v) of DMSO were used as a control. After 16 h of incubation at 25°C, the spore germinated of *Foc* TR4 conidia were counted using a light microscope (Axio Scope A1, Carl ZEISS, Germany). Conidia were considered as germinated when germ tube began to appear. Five replicates were used in each treatment and at least 200 conidia were measured per replicate.

### Effect of Extract on Mycelial Morphology and Ultrastructure of *Foc* TR4

*Foc* TR4 was grown on the PDA medium with 4 × EC_50_ of extract at 28°C for 5 days. The collected mycelia were fixed with 2.5% (v/v) of glutaraldehyde overnight at 4°C. DMSO (10%, v/v) treatment was used as a control. Agar plugs (5 mm in diameter) with *Foc* TR4 were cut from the edge of a 3-day-old fungal medium. The mycelial sections were prepared according to Jing et al. (2020). Morphological characteristics of *Foc* TR4 mycelia were detected using SEM. The effect of strain 8ZJF-21 extract on the cellular ultrastructure of *Foc* TR4 was observed by a transmission electron microscope (TEM, JEM-1400 Flash, Hitachi Limited, Tokyo, Japan) according to Wei et al. (2020). Four replicates were used per treatment and each experiment was repeated three times.

### Component Identification of Strain 8Zjf-21 Extract by Gas Chromatography-Mass Spectrometry

The volatile organic compounds in strain 8ZJF-21 extract were identified using GC-MS as our previous description (Li et al., 2021). Extract of strain 8ZJF-21 was first dissolved in the chromatographic grade methanol and was filtered through a 0.2-μm filter. The solution was injected into a gas capillary column (DB-FFAP, 30 m × 0.25 mm × 0.25 μm) of a gas chromatograph (5973 Inert XL MSD, Agilent, United States). Helium was used as a carrier gas with a flow rate of 1 ml min^–1^. The column temperatures were set as follows: initial column temperature at 70°C for 3 min, followed by an increment of 5°C/min up to 100°C and 10°C/min up to 250°C. The final temperature was kept at 300°C for 5 min. The mass spectrometer was operated in the electron ionization mode at 70 eV with a continuous scan from 50 to 800 m/z (Jing et al., 2020). The peaks were identified by matching the mass spectra with the National Institute of Standards and Technology (NIST, United States) library.

### Statistical Analysis

All the experiments were implemented using a completely randomized design. Data were obtained from at least three biological replicates and were expressed as the mean ± standard deviation (SD). Data processing and statistical analysis were performed with the SPSS statistical software package (SPSS Inc., Cary, NC, United States, v.22). The significance was determined by Duncan’s multiple range tests (*P* < 0.05).

## Results

### Morphological, Biochemical, and Physiological Characteristics of Strain 8ZJF-21

**FIGURE 1:**
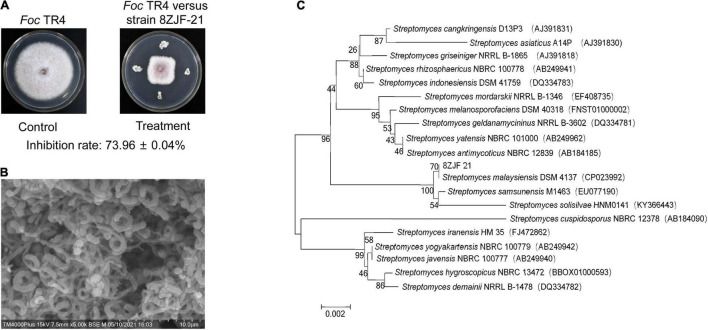
Antifungal activity and identification of strain 8ZJF-21. **(A)** Antifungal activity of strain 8ZJF-21 on mycelial growth of *Foc* TR4. **(B)** Morphology of strain 8ZJF-21. **(C)** Phylogenetic tree of strain 8ZJF-21 using 16 rDNA sequences. The tree was constructed using the NJ method in the MEGA software. The level of bootstrap support (1,000 repetitions) was indicated at all nodes.

Antifungal activity of the selected endophytic actinomycetes was further tested against *Foc* TR4. Strain 8ZJF-21 isolated from the roots of medicinal plant *C. capitulata* exhibited a strong antagonistic activity (Figure 1A). Strain 8ZJF-21 can grow well on PDA and various ISP media (ISP2-ISP7). Different morphological characteristics of the colony were displayed in Supplementary Table 2. The strain can develop the branched substrate and aerial mycelia. Cream aerial mycelia and milky-white substrate hyphae were observed on all the selected ISP media. The grayish-white color of aerial and substrate mycelia was displayed on PDA. Diffusible pigment was not detected on the selected media except for ISP7, which was a typical profile of melanin pigment produced by *Streptomyces* (Shirling and Gottlieb, 1966). The spiral spore chains with rough surface were finally generated (Figure 1B).

In comparison with different culture conditions in the ISP2 medium, strain 8ZJF-21 can grow well at temperatures from 20 to 40°C (optimum at 30°C), NaCl up to 3% (w/v, optimal concentration 1%) and pH from 6.0 to 9.0 (optimum pH 7.0). It could produce extracellular enzymes such as amylase, cellulase, protease, and urease as well as reduce nitrate. It had no capacity to produce H_2_S and respond to gelatin degradation. In addition, strain 8ZJF-21 was able to utilize all the tested sugars as sole carbon source and most of nitrogen source except for NH_4_NO_3_, arginine, and glutamic acid (Table 1). Compared with the reference *Streptomyces* strains (Qi et al., 2019; Jing et al., 2020; Wei et al., 2020), strain 8ZJF-21 was considered as a member of the genus *Streptomyces*.

**TABLE 1:** Determination of physiological and biochemical properties of *S. malaysiensis* 8ZJF-21.

	Result	Characteristics	Result
**Biochemical test**		**Carbon source**	
Tween-20	−	Raffinose	+
Tween-40	−	D-Trehalose anhydrous	+
Tween-80	−	α-Lactose	+
Gelatin Liquefaction	−	Inositol	+
Starch hydrolysis	+	D(+)-Cellobiose	+
IAA production	+	D-Fructose	+
Cellulase	+	D-Melezitose	+
Urease	+	L-Arabinose	+
Protease	+	Ribose	+
Lipase	+	D-Galactose	+
H_2_S production	−	D-Glucose	+
Nitrate reduction	+		
Siderophores	+		
pH tolerance test	6–9 (optimal pH 7.0)		
Temperature tolerance test	20°C–40°C (optimum at 30°C)		
NaCl tolerance test(%)	< 3 (optimal NaCl concentration 1%)		
**Nitrogen source**			
NH_4_Cl	+	Histidine	+
(NH_4_)_2_SO_4_	+	Tyrosine	+
NH_4_NO_3_	−	Methionine	+
Arginine	−	Glutamic acid	−
Glycine	+	Hydroxyproline	+
Phenylalanine	+		

*“+” positive result; “−” negative result.*

### Identification of Strain 8ZJF-21

To further identify strain 8ZJF-21, the whole genome was sequenced. A 1,639 bp-length sequence of 16S rDNA was extracted from the genome sequences. One-hundred percent of nucleotide similarity was found with 16S rDNA of *S. malaysiensis* DSM 4137 (GenBank ID: NZ_CP023992). The phylogenetic tree showed that strain 8ZJF-21 was located in a well-delineated subclade with *S. malaysiensis* (Figure 1C). Compared with the genomes of strain 8ZJF-21 to the typal genome of *S. malaysiensis* DSM 4137 (Supplementary Figure 1), the calculated ANI value was 98.49 above the threshold value of 95-96% for species delineation (Richter and Rosselló-Móra, 2009). Therefore, strain 8ZJF-21 was identified as *S. malaysiensis*.

### Detection of a Broad-Spectrum Antifungal Activity of *Streptomyces malaysiensis* 8ZJF-21 Against Phytopathogenic Fungi

**FIGURE 2:**
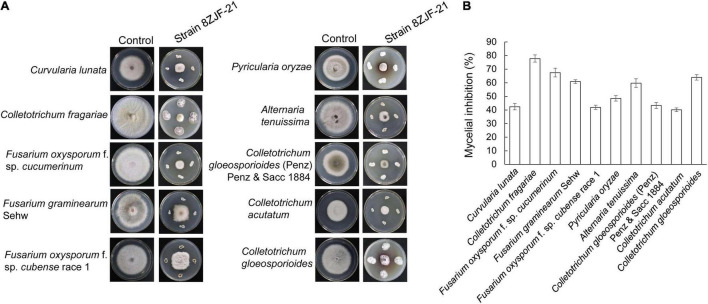
Measurement of a broad-spectrum antifungal activity of *S. malaysiensis* 8ZJF-21 against the selected fungal phytopathogens. **(A)** Antagonistic assay of *S. malaysiensis* 8ZJF-21 against different phytopathogens. **(B)** Quantitative analysis of antifungal activity of *S. malaysiensis* 8ZJF-21 against different phytopathogens.

The antifungal assay showed that *S. malaysiensis* 8ZJF-21 significantly inhibited the mycelial growth of all tested fungi (Figure 2). The inhibition rates ranged from 40.15 to 77.83%. The strongest antifungal activity was detected against the causal agent of strawberry anthracnose (*C. fragariae*, 77.83 ± 2.68), followed by *F. oxysporum* f. sp. *cucumerinum* (67.48 ± 1.32), *C. gloeosporioides* (63.96 ± 2.01), and *F. graminearum* Sehw (60.86 ± 1.32). In addition, strain 8ZJF-21 had also strong inhibition activities against *A. tenuissima* (59.73 ± 3.2), *C. lunata* (42.37 ± 2.31), *F. oxysporum* f. sp. *cubense* race 1 (42.88 ± 1.58), *C. gloeosporioides* (Penz) Penz and Sacc 1884 (43.33 ± 2.18), *P. oryzae* (48.51 ± 2.01), and *C. acutatum* (40.15 ± 1.32). It suggested that *S. malaysiensis* 8ZJF-21 had a broad-spectrum antifungal activity.

### Biocontrol of *Foc* TR4 and Plant-Growth Promoting in the Pot Experiment

**FIGURE 3:**
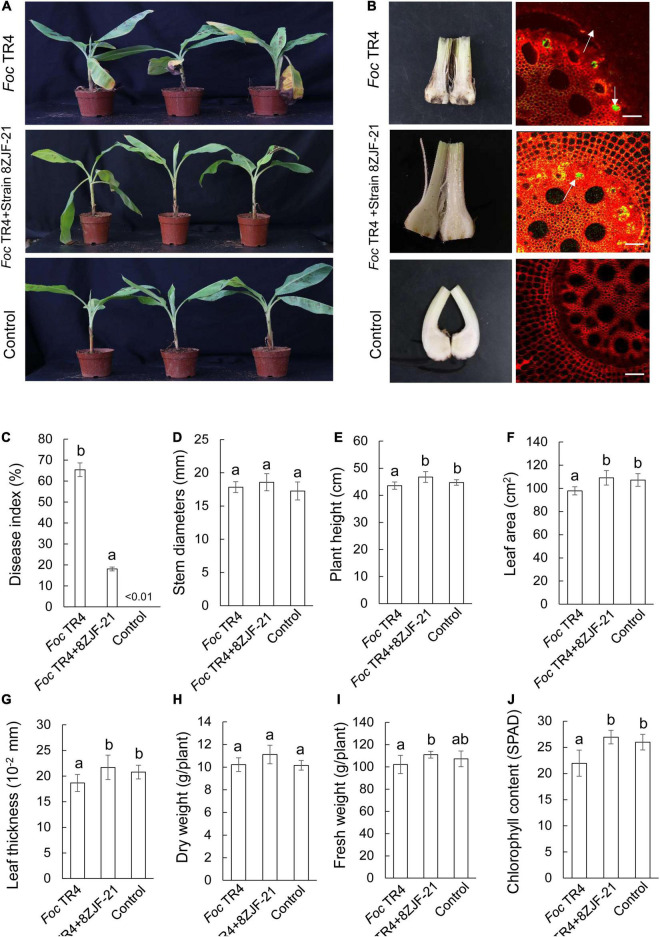
Assay of Fusarium wilt disease control and plant-growth promoting after treatment with *S. malaysiensis* 8ZJF-21. **(A)** Chlorotic symptom of leaves in different treatment groups at 30 dpi. **(B)** Detection of Foc TR4 infection in the corm and root of banana seedlings at 30 dpi. **(C)** Quantitative analysis of disease index of banana seedlings at 30 dpi. Determination of physiological indicators including stem diameter **(D)**, plant height **(E)**, leaf area **(F)**, leaf thickness **(G)**, dry weight **(H)**, fresh weight **(I)**, and chlorophyll content **(J)** in different treatment groups at 30 dpi. Error bars indicate standard errors of the means from three repeated experiments. Different letters indicate a significant difference according to Duncan’s multiple range test (*P* < 0.05).

*Streptomyces malaysiensis* 8ZJF-21 inhibited the growth of different phytopathogenic fungi *in vitro*. It promoted us to evaluate its biocontrol efficiency against *Foc* TR4 using the pot experiment. The disease symptoms on banana seedlings were detected at 30 dpi. In the treatment group of *Foc* TR4 (G1), banana seedlings showed an obvious chlorotic symptom at the bottom of the leaves (Figure 3A). Compared with the control group, no obvious disease symptom was detected in the group of *S. malaysiensis* 8ZJF-21 + *Foc* TR4 (G2), suggesting that the protective treatment with *S. malaysiensis* 8ZJF-21 effectively prevented the infection of *Foc* TR4. The results were supported by the lack of obvious black symptoms in the split corms of banana seedlings treated with *S. malaysiensis* 8ZJF-21 (G2). We also evaluated *Foc* TR4-GFP infection in the roots of banana seedlings. The colony-forming units of *Foc* TR4-GFP in *S. malaysiensis* 8ZJF-21-treated roots were much lower than that in *Foc* TR4-GFP-treated roots (Figure 3B). The disease index was 65.37% in the G1 group, while only 18.07% were recorded in the G2 group (Figure 3C).

Compared to different agronomic traits of banana seedlings in different treatment groups (Figures 3D–J), *Foc* TR4 infection inhibited the growth of banana seedlings. Although no obvious difference of stem diameter was observed among the three treatment groups (Figure 3D), *S. malaysiensis* 8ZJF-21 significantly increased (*p* = 0.0036) the plant height and reached 46.73 ± 2.01 cm at 30 dpi (Figure 3E). Compared to the agronomic indicators in the G1 group, a significant increase was detected in the leaf area, leaf thickness, dry weight, and fresh weight in the G2 and G3 groups (Figures 3F–I). Chlorophyll content was sharply decreased in the *Foc* TR4-treated leaves due to chlorotic symptom (Figure 3I). Hence, *S. malaysiensis* 8ZJF-21 not only reduced the disease symptoms, but also promoted the growth of banana seedlings.

### Effect of Extract on the Antioxidant System of Banana Seedlings

**FIGURE 4:**
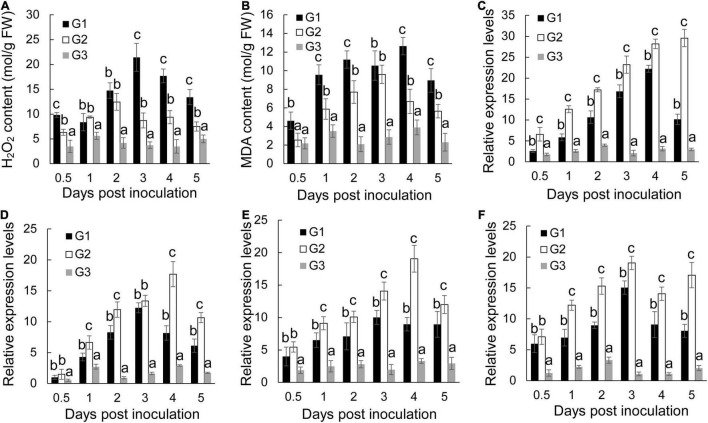
Effects of *S. malaysiensis* 8ZJF-21 on the activation of antioxidant system. G1: *Foc* TR4-GFP treatment; G2: *S. malaysiensis* 8ZJF-21 + *Foc* TR4-GFP treatment; G3: medium treatment. **(A)** Measurement of H_2_O_2_ content in the roots of banana seedlings after treatment at different time points. **(B)** Measurement of MDA content in the roots of banana seedlings after treatment at different time points. The expression levels of antioxidant enzyme genes were determined by qRT-PCR, including *MaCAT* **(C)**, *MaSOD* **(D)**, *MaPOD* **(E)**, and *MaPPO* **(F)**. Error bars indicate standard errors of the means from three repeated experiments. Different letters indicate a significant difference according to Duncan’s multiple range test (*P* < 0.05).

Biotic and abiotic stresses induce accumulation of reactive oxygen species in plant cells, thereby causing oxidative damage (Li et al., 2015). The oxidative damage expressed as the form of H_2_O_2_ was first determined in banana roots of different groups. *Foc* TR4 infection resulted in a rapid increase of H_2_O_2_ and reached a peak at 3 dpi (Figure 4A). *S. malaysiensis* 8ZJF-21 reduced the accumulation of H_2_O_2_ in *Foc* TR4-infected roots. It was supported by the changes of MDA contents in the G2 group, a marker for monitoring lipid peroxidation caused by oxidative damage (Figure 4B). The MDA contents in *Foc* TR4-inoculated roots dramatically increased from 0.5 dpi and reached the highest value at 4 dpi, which was four-fold higher than that in the G3 group. However, the increase of MDA contents in roots treated with *S. malaysiensis* 8ZJF-21 was obviously inhibited. The maximum was detected at 4dpi with the decrease of 69.04% in comparison with the G1 group.

To assay whether *S. malaysiensis* 8ZJF-21 could induce activities of antioxidant enzymes (such as CAT, SOD, PPO, and POD), the expression levels of these genes were investigated. The transcripts of *MaCAT* in banana roots treated with *S. malaysiensis* 8ZJF-21 increased gradually until 5 dpi (Figure 4C). In *Foc* TR4-treated roots, the expression peak was detected at 4 dpi. No obvious increase (*P* < 0.01) among different time points except for 2 dpi was detected in the G3 group. Similarly, *S. malaysiensis* 8ZJF-21 upregulated significantly (*P* < 0.05) the expression levels of *MaSOD* and *MaPOD*. Their transcripts reached the maximum at 4 dpi with two-fold higher than those in the G1 group (Figures 4D,E). Although *Foc* TR4 induced obviously the transcript accumulation of *MaPPO*, the expression levels were higher in the G2 group and increased by 21% at 3 dpi in comparison with *Foc* TR4-treated roots (Figure 4F).

### Expression Levels of Defense-Related Genes in Banana Roots Treated With *Streptomyces malaysiensis* 8ZJF-21

**FIGURE 5:**
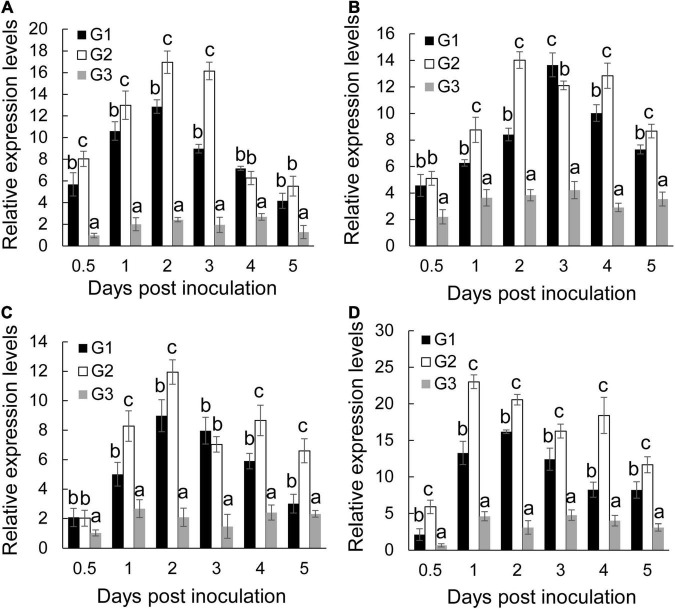
Expression analysis of defense-related marker genes in banana roots. qRT-PCR determined the transcript levels of *MaPR1* **(A)**, *MaPAL* **(B)**, *Mab-1,3-Glu* **(C)**, and *MaMAPK1* **(D)** in banana roots after treatment with *S. malaysiensis* 8ZJF-21 at different time points. G1-G3 represented different treatment groups as described in Figure 4. Error bars indicate standard errors of the means from three repeated experiments. Different letters indicate a significant difference according to Duncan’s multiple range test (*P* < 0.05).

To determine whether the defensive system was activated in response to *S. malaysiensis* 8ZJF-21 and/or *Foc* TR4, four defense-related genes (*Mab-1,3-Glu*, *MaPAL*, *MaMAPK1*, and *MaPR1*) were selected. By contrast, *S. malaysiensis* 8ZJF-21 and/or *Foc* TR4 significantly increased the transcripts of four defense-related genes with varying patterns (Figure 5). The transcript level of *MaPR1* in roots treated with *S. malaysiensis* 8ZJF-21 increased significantly at 0.5 dpi and reached the highest peak at 2 dpi, which was 1.3-fold and 7.0-fold higher than that in the G1 and G3 groups, respectively (Figure 5A). The transcript level of *MaPAL* was also upregulated by *S. malaysiensis* 8ZJF-21 and the expression peak was detected at 2 dpi with 1.6-fold higher than that in the G1 group (Figure 5B). A similar expression pattern of *Ma*β*-1,3-Glu* was observed in the treated roots of *S. malaysiensis* 8ZJF-21. The transcripts of *Ma*β*-1,3-Glu* in the G2 group showed an increase of 1.64-fold at 1 dpi and 1.35-fold at 2 dpi in comparison with that in the G1 group (Figure 5C). The expression levels of *MaMAPK1* reached their maximum values at 2 dpi in the G1 group and at 1 dpi in the G2 group. High transcripts were maintained by *S. malaysiensis* 8ZJF-21 until 4 dpi (Figure 5D). It suggested that *S. malaysiensis* 8ZJF-21 could improve the plant resistance to *Foc* TR4 by activating the MAPK-mediated signaling pathway of defense response.

### Effect of Extract on the Growth of *Foc* TR4

**FIGURE 6:**
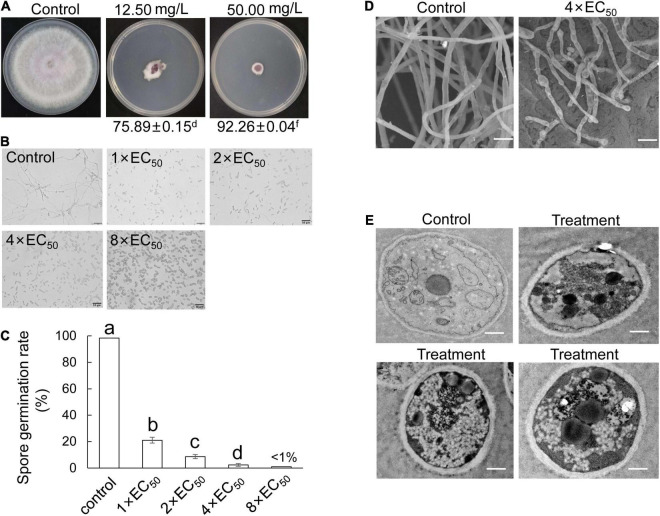
Antifungal mechanism of *S. malaysiensis* 8ZJF-21 extract on *Foc* TR4 *in vitro*. **(A)** Inhibition ability assay of different dose extracts on the hyphal growth of Foc TR4. **(B)** Inhibition efficiency of different dose extracts on spore germination of *Foc* TR4. Bar = 10 μm. **(C)** Quantitative analysis of spore germination of *Foc* TR4 after treatment with different dose extracts. Different letters indicate a significant difference according to Duncan’s multiple range test (*P* < 0.05). **(D)** Characteristics of hyphal morphology of *Foc* TR4 after treatment with 4 × EC_50_ extract. Bar = 1 μm. **(E)** Ultrastructural characteristics of *Foc* TR4 after treatment with 4 × EC_50_ extract. Bar = 0.5 μm.

*Foc* TR4 was inoculated on the PDA plate containing different concentration extracts of *S. malaysiensis* 8ZJF-21. The inhibition of mycelial growth was measured, until hypha reached the edge of the plate in the control group (10% of DMSO treatment). The mycelial growth of *Foc* TR4 was inhibited dramatically along with the increase of extract concentration. More than 12.50 μg/ml of extract almost completely restricted the mycelial growth of *Foc* TR4 (Figure 6A). The EC_50_ value was 6.11 μg/ml (Supplementary Figure 2). Similarly, the extracts significantly reduced the germination rate of conidia and the length reduction of germ tubes (Figures 6B,C). All spore germination was almost completely inhibited by 4 × EC_50_ of extract. No obvious inhibition of *Foc* TR4 growth and spore germination was observed in the control group. In addition, the extract-treated hyphae became deformed, shrunk, ruptured, and swollen (Figure 6D). The normal hyphae with a smooth surface appeared to be uniform in thickness. For cellular ultrastructure of *Foc* TR4, 4 × EC_50_ of the extract caused vacuolization and organelle degradation. Mitochondria and cell nucleus gradually disappeared. High dense components were formed in treated cells (Figure 6E).

### Genome Sequencing and Annotation of *Streptomyces malaysiensis* 8ZJF-21

**FIGURE 7:**
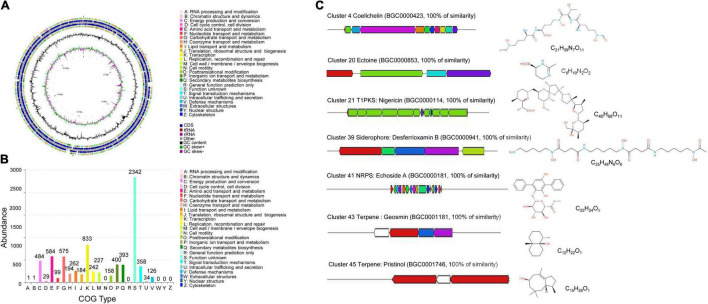
Genome annotation of *S. malaysiensis* 8ZJF-21 and BGC prediction. **(A)** Circular map of strain 8ZJF-21 genome. From outside to center, ring 1 and ring 4 represented CDS in forward strand and reverse strand, respectively. Different colors indicate the functional category of COG. Rings 2 and 3 showed the CDS, tRNA, and rRNA in forward strand and reverse strand, respectively. Ring 5 and ring 6 show the G + C content and G + C skew, respectively. **(B)** COG functional annotation of *S. malaysiensis* 8ZJF-21 genome. **(C)** Genomic information and chemical structure of BGCs with 100% similarity with the known BGCs.

After sequencing and assembly, the genome of *S. malaysiensis* 8ZJF-21 consisted of 11,434,537 bp and had 71.09% of GC content. The genome contained 8 rRNA genes, 63 tRNA genes, and 9,787 coding sequences (Figure 7A). By annotation, 34.24, 48.33, and 75.44% of genes were assigned to three categories of KEGG, GO, and COG, respectively. In KEGG annotation, 2,491 of genes participated into the regulation of cellular processes (193), metabolism (2,008), human diseases (142), genetic information processing (216), environmental information processing (298), and organismal systems (44) (Supplementary Figure 3). For COG annotation, the top five categories contained transcription (833), nucleotide transport and metabolism (584), carbohydrate transport and metabolism (575), energy production and conversion (484) as well as inorganic ion transport and metabolism (400). Notably, 2,342 of genes were clustered into unknown function category (Figure 7B). A total of 4,730 genes were annotated into biological process (1,728), cellular component (1,657), and molecular function (3,950) using the GO database (Supplementary Figure 4).

Moreover, several BGCs in the genome of *S. malaysiensis* 8ZJF-21 were involved in secondary metabolism. By alignment with antiSMASH, the predicted 52 BGCs included NRPS (non-ribosomal peptide synthetase), PKS (polyketide synthase) type 1 and 2, siderophore, terpene, indole, butyrolactone, and betalactone (Supplementary Table 3). Fourteen BGCs exhibited more than 70% of similarity with the submitted BGCs in the database. Seven BGCs showed more than 100% of similarity with coelichelin, ectoine, nigericin B, ectoine, desferrioxamin B, echoside A/B, geosmin, and pristinol (Figure 7C). Six BGCs participated in the biosynthesis of antimicrobial compounds such as hopene, elaiophylin, coelichelin, ectoine, nigericin, and geldanamycin. Two BGCs probably regulated the biosynthesis of anticancer agent (hygrocin A/B and azalomycin F3a). Two siderophore molecules were encoded by cluster 21 and cluster 39. Gene clusters 67 and 78 were responsible for the biosynthesis of terpene. Clusters 66 and 71 were involved in the production of pigment. Notably, a high portion of unknown BGCs suggests that several novel secondary metabolites could be produced by *S. malaysiensis* 8ZJF-21.

### Component Identification of Strain 8ZJF-21 Extract by Gas Chromatography-Mass Spectrometry

Gas Chromatography-Mass Spectrometry (GC-MS) was used to identify the bioactive compounds in *S. malaysiensis* 8ZJF-21 extract. Compared to mass spectra with the NIST library, 19 volatile organic compounds were obtained according to retention time and molecular weight (Table 2). They contained acetophenone (1), chloroacetic acid, 3-tetradecyl ester (2), 2,4-furandicarboxylic acid, dimethyl ester (3), formic acid, trans-4-methylcyclohexyl ester (4), 5-hydroxymethylfurfural (5), cyclohexane (6), pyrazoline (7), pentacosanoic acid, methyl ester (8), hexadecanoic acid, ethyl ester (9), borneol, dimethyl(pentafluorophenyl)silyl ether (10), pentacosanoic acid, methyl ester (11), 1,2-benzenedicarboxylic acid, butyl 2-methylpropyl ester (12), 4-(3-methyl-2-butenyl)-1H-indole (13), 1,2-bis(p-(cis-styryl)phenyl)-trans-ethylene (14), colchiceinamide (15), 1,2-Bis(p-(cis-styryl)phenyl)-trans-ethylene (16), voaluteine (17), 4’-(3-(6-Methyl-3-pyridyl)-1-(p-tolyl)-2-pyrazolin-5-yl)acetanilide (18), and bufotalin (19). The peak area represented the relative proportion. Benzenedicarboxylic acid and 1H-Indole, 4-(3-methyl-2-butenyl) were two dominant components in *S. malaysiensis* 8ZJF-21 extract.

**TABLE 2:** Identification of compound components in *S. malaysiensis* 8ZJF-21 extract by GC-MS.

No.	Retention time (min)	Peak area (Ab*s)	Baseline height (Ab)	Absolute height (Ab)	Peak width 50% (min)	Compounds	Molecular weight (amu)
1	13.12	70692	10852	19633	0.201	Acetophenone	120.058
2	14.294	18901	6915	14847	0.101	Chloroacetic acid	290.201
3	23.144	225670	46063	61558	0.268	2,4-Furandicarboxylic acid	184.037
4	24.679	249440	63347	86147	0.176	Formic acid	142.099
5	25.183	439371	113658	138820	0.159	5-Hydroxymethylfurfural	126.032
6	25.384	90802	21662	53877	0.159	Cyclohexane	112.125
7	25.594	1735455	503739	535781	0.201	Pyrazoline	112.100
8	26.802	88591	19037	55004	0.151	Pentacosanoic acid	396.397
9	28.085	152797	32432	77364	0.168	Hexadecanoic acid	284.272
10	29.377	83552	29050	90809	0.109	Dimethyl(pentafluorophenyl) silyl ether	378.144
11	30.971	342717	70703	162678	0.185	Hexadecanoic acid	284.272
12	37.782	2562283	756038	1177089	0.151	Benzenedicarboxylic acid	278.152
13	38.093	2371047	670528	1123216	0.185	4-(3-methyl-2-butenyl)-1H-indole	185.12
14	38.948	205896	48409	565497	0.143	1,2-Bis(p-(cis-styryl)phenyl)-trans-ethylene	384.188
15	40.551	474633	71382	760234	0.243	Colchiceinamide	384.169
16	40.836	524589	86333	811105	0.294	1,2-Bis(p-(cis-styryl)phenyl)-trans-ethylene	384.188
17	42.337	893803	104279	1086622	0.428	Voaluteine	384.205
18	43.31	901704	45557	1234395	0.738	4′-(3-(6-Methyl-3-pyridyl)-1-(p-tolyl)-2-pyrazolin-5-yl)acetanilide	384.195
19	43.956	542604	79901	1440011	0.252	Bufotalin	444.251

## Discussion

Actinobacteria are an important component of soil microbial communities, accounting for around 10% of the total soil microbiome (van Bergeijk et al., 2020). Some of them can enter directly into plant tissues and establish an endophytic lifestyle (Dinesh et al., 2017). Endophytic actinomycetes from medicinal plants were reported as major sources of antifungal agents (Golinska et al., 2015). However, there is still a lack of knowledge on their properties and application in the field. It prompted us to explore endophytic *Streptomyces* from medicinal plants as biocontrol agents. Our previous study demonstrated that 144 endophytic Actinomycete strains were isolated from different tissues of traditional medicinal plants. Especially, *Streptomyces* sp. strain 8ZJF-21 isolated from the roots of *C. capitulata* exhibited a strong antifungal activity against *Foc* TR4. Accumulated evidence indicated that secondary metabolites of medicinal plants promoted the development of microbial traits by mediating cross-talk between endophytes and their hosts (Granér et al., 2003). During the long-term interaction, endophytes gained some new genetic information and produced specific bioactive compounds (Chithra et al., 2014). Some rare actinomycetes isolated from the medicinal plant *Vochysia divergens* produced a wide diversity of antibacterial secondary metabolites (Gos et al., 2017). Our present results also showed that an endophytic *Streptomyces* sp. strain 8ZJF-21 exhibited strong antagonistic activities against *Fusarium* spp., *Curvularia* spp., *Alternaria* spp., and *Pyricularia* spp. (Figure 2). Similarly, 12 out of 65 endophytic actinomycetes isolated from medicinal plants *Artemisia argyi*, *Paeonia lactiflora*, *Radix platycodi*, and *Achyranthes bidentata* effectively suppressed penicillin-resistant *Staphylococcus aureus*, and majority of them belonged to *Streptomyces* (Zhang et al., 2012). Therefore, endophytic actinomycetes from medicinal plants could be exploited as a novel source of biocontrol agents.

Until now, a number of endophytic *Streptomyces* species were isolated from different plant tissues, but many of which were poorly defined. In our study, the morphological, physiological, and biochemical characteristics of strain 8ZJF-21 were consistent with the properties of the genus *Streptomyces*. The phylogenetic tree of 16S rDNA showed that the strain clustered into the same subgroup with *S. malaysiensis* DSM 4137. Sharma and Manhas (2020) reported that an obvious difference of morphological characteristics was found among the nearest phylogenetic relative strains. Hence, 16S rDNA did not provide a sufficient resolution for the species-level identification. Although the traditional method of DNA-DNA hybridization allowed the classification for prokaryote, serious shortcomings limited its application such as a time-consuming procedure, operational feasibility, and standard stain obtaining (Gevers et al., 2005). Based on the sequencing genomes, ANI provided an efficient method to identify the level of species (Gevers et al., 2005). It was supported by an ANI value of 98.49% that was calculated by the genomic alignment of strain 8ZJF-21 with the typal strain *S. malaysiensis* DSM 4137, which was above the threshold value of 95–96% for species delineation (Richter and Rosselló-Móra, 2009).

To further evaluate the biocontrol efficiency of *S. malaysiensis* 8ZJF-21, the pot experiment was carried out in this study. Strain 8ZJF-21 improved the system resistance of banana seedlings and inhibited the infection of *Foc* TR4. The previous reports also showed that the plant immune system could be triggered after the inoculation with pathogen or beneficial microbes (Zhang et al., 2019). In an early response, H_2_O_2_ is a key signaling molecule in early plant immune responses (Li et al., 2015). *Foc* TR4 induced H_2_O_2_ outbreak and MDA accumulation in banana roots. Lower levels of H_2_O_2_ were detected in roots treated with *S. malaysiensis* 8ZJF-21, suggesting that the strain alleviated the oxidative stress caused by *Foc* TR4. Moreover, *S. malaysiensis* 8ZJF-21 induced the higher and lasting expression levels of antioxidant enzyme genes (*MaPPO*, *MaPOD*, *MaCAT*, and *MaSOD*) and defense-related genes (*MaPAL*, *MaPR-1*, *MaMAPK1*, and *Ma*β*-1,3 glu*). PPO could oxidize phenol and transform phytoalexins to enhance plant resistance to pathogens (Richter et al., 2012). PAL degraded phenylalanine to trans-cinnamic acid, activating the biosynthesis of salicylic acid (SA) to induce the defense response (Shine et al., 2016). Hence, the SA-dependent signaling pathway might participate in the resistance activation of *S. malaysiensis* 8ZJF-21. Similarly, *Pseudomonas putida* and *Pseudomonas syringae* stimulated a systemic response against *Alternaria solani* by increasing the activities of PAL, POD, and PPO (Ahmed et al., 2011). *Streptomyces goshikiensis* triggered defense response against *Fusarium oxysporum* f. sp. *niveum* by enhancing activities of PPO, SOD, and β-1,3 glucanases (Faheem et al., 2015). Our previous studies also revealed that *Streptomyces* can activate defensive enzyme activities and inhibit the infection of *Foc* TR4 in banana roots (Zhang et al., 2021). Therefore, the expression of defense-related and defensive enzyme genes was associated with the priming of antagonistic microbes on host plants as an early and rapid response to pathogens.

Additionally, the metabolites of *S. malaysiensis* 8ZJF-21 exhibited strong antifungal activity against *Foc* TR4. The extract directly attacked fungal pathogens, resulting in abnormal morphology like sporulation inhibition, swollen and distorted mycelia, vacuolation, and organelle disappearance. Moreover, endophytic actinomycetes produced a large set of metabolic compounds to stimulate the expression of specific genes involved in resistance to pathogens (Taechowisan et al., 2005; Kenneth et al., 2019; van Bergeijk et al., 2020). These metabolites also had a strong influence on the rhizosphere colonization of endophytic actinomycetes (Zhang et al., 2021). Competition of nutrient availability and niche was an essential for biocontrol among pathogenic and non-pathogenic microbes (Heydari and Pessarakli, 2010). Our previous study reported that competitive colonization of *Streptomyces* sp. BITDG-11 reduced fungal population of *Foc* TR4 in banana roots (Zhang et al., 2021). The biocontrol agents depleted rapidly the limited nutrient making it unavailable to meet the growth of pathogens. It is noteworthy that *S. malaysiensis* 8ZJF-21 can also promote the growth of banana seedlings. Its production ability of siderophores, cellulose, and IAA supported the physiological characteristics of plant-growth promoting. Similar results were reported that endophytes promoted plant growth by producing siderophores, decomposing organic materials by cellulose or lignocellulose and also producing growth promoters such as IAA and gibberellic acid (Taechowisan et al., 2005). They formed a symbiotic relationship with the host plants, facilitating plant to uptake nutrients from the soil (Rosenblueth and Martínez-Romero, 2006). The nutrient cycling capacity made them ideal candidates for natural fertilizers. Therefore, the endophytic actinomycetes will be potential biocontrol agents against plant diseases caused by soil-borne pathogens and plant growth promoters.

To identify fully the biosynthetic potential of secondary metabolites, the genome of *S. malaysiensis* 8ZJF-21 was sequenced and annotated. Fifty-two BGCs were predicted for producing known or unknown secondary metabolites, including terpenes, PKS type I or type II, NRPS, siderophores, and ectoines. PKS and NRPS were mainly responsible for the synthesis of most biologically active polyketide and peptide compounds (Janso and Carter, 2010; Qi et al., 2019). Especially, BGCs of desferrioxamin B, coelichelin, ectoine, nigericin, echoside A, geosmin, and pristinal showed 100% similarity with known structures. Desferrioxamines B and coelichelin belonged to different types siderophores. Siderophore produced by *Streptomyces* spp. played a crucial role in suppressing *Fusarium* wilt disease by depleting iron (Vurukonda et al., 2018; Zeng et al., 2018; Zhang et al., 2021). Other siderophore-producing rhizobacteria were also reported as biocontrol agents including *Pseudomonas koreensis*, *Burkholderia cepacian*, *Rahnella aquatilis*, and *Bacillus subtilis* (Carmona-Hernandez et al., 2019; Ghazy and El-Nahrawy, 2021). Ectoine could interact with biomolecules such as lipids, proteins, and DNA to protect itself from environmental stresses (Fenizia et al., 2020; Wittmar et al., 2020). Nigericin produced by endophytic *S. endus* OsiSh-2 exhibited remarkable antagonistic activity against rice blast disease (Xu et al., 2017). Echoside A from *Streptomyces* sp. GMR22 had a high potential as an antiviral agent (Melinda et al., 2021). Pristinol was also identified as a sesquiterpene alcohol from *S. pristinaespiralis* (Klapschinski et al., 2016). In addition, the predicted cluster 11 containing 38 genes showed 96% of similarity with BGCs of hygrocin A/hygrocin B. Hygrocins belonging to a type of naphthoquinone ansamycins had antitumor and antimicrobial activities (Wang et al., 2018). Cluster 19 exhibited 95% of similarity with BGC of azalomycin F3a. Azalomycin F and its analogs from different *Streptomyces* strains had broad-spectrum antimicrobial activities (Yuan et al., 2013). Notably, much more unknown BGCs were identified, suggesting that *S. malaysiensis* 8ZJF-21 had a great potential for producing novel secondary metabolites. It was supported that 33.9% of coding genes clustered into the unknown function category in the COG annotation (Figure 7B). How a number of PKS and NRPS gene clusters regulate the biosynthesis of bioactive metabolites still needs to be further investigated.

Gas chromatography-mass spectrometry was used to further identify the antifungal compounds of *S. malaysiensis* 8ZJF-21 extract in our study. A large number of acid compounds such as chloroacetic acid, furandicarboxylic acid, formic acid, pentacosanoic acid, hexadecanoic acid, and benzenedicarboxylic acid were a main type of antifungal production. Benzenedicarboxylic acid possessing high peak area is a main metabolite of *S. cuspidosporus* with high antagonistic activity against pathogenic bacteria, fungi, and nematode (Sholkamy et al., 2020). Chloroacetic acid and hexadecanoic acid had the potential for controlling *Colletotrichum gloeosporioides* (Rajaofera et al., 2019). In addition, 1H-Indole, 4-(3-methyl-2-butenyl) was the other main component in *S. malaysiensis* 8ZJF-21 extract. The compound produced by *Aeromonas hydrophila* was highly effective to suppress the growth of *Aspergillus flavus* (Mannaa and Kim, 2018). Thus, these compounds could altogether contribute to the broad-spectrum antifungal activity of *S. malaysiensis* 8ZJF-21. Interestingly, BGCs of these compounds were not found in its genome. It might be because of the different identification methods and alignment databases (Wei et al., 2020).

## Conclusion

In the study, an endophytic strain 8ZJF-21 with strong antifungal activity was identified from the roots of a medicinal plant. Based on the morphological, physiological, and biochemical characteristics, the strain was defined as the genus *Streptomyces*. The phylogenetic tree and ANI calculation were further used to identify strain 8ZJF-21 as *S. malaysiensis*. The pot experiment demonstrated that *S. malaysiensis* 8ZJF-21 improved plant resistance against *Foc* TR4 and promoted the growth of banana seedlings. The antifungal mechanism showed that *S. malaysiensis* 8ZJF-21 extract could inhibit the spore germination and mycelial growth of *Foc* TR4, and damage the ultrastructure of pathogenic cells. Genome annotation and GC-MS analysis revealed that strain 8ZJF-21 has a great potential for producing bioactive metabolites, suggesting that it will become an essential biocontrol agent against *Foc* TR4.

## Data Availability Statement

The datasets presented in this study can be found in online repositories. The names of the repository/repositories and accession number(s) can be found in the article/Supplementary Material.

## Author Contributions

LZ, ZL, and WW developed the ideas and designed the experimental plans. LZ and WW supervised the research, provided the fund support, and prepared the manuscript. LZ, YW, SW, and YH performed the experiments. YH, TY, and JX provided the materials. LZ, YW, JZ, TY, and WW analyzed the data. All authors contributed to the article and approved the submitted version.

## Conflict of Interest

The authors declare that the research was conducted in the absence of any commercial or financial relationships that could be construed as a potential conflict of interest.

## Publisher’s Note

All claims expressed in this article are solely those of the authors and do not necessarily represent those of their affiliated organizations, or those of the publisher, the editors and the reviewers. Any product that may be evaluated in this article, or claim that may be made by its manufacturer, is not guaranteed or endorsed by the publisher.

## Footnotes

1
www.majorbio.com

2
http://www.ezbiocloud.net/eztaxon/identify

3
https://www.ezbiocloud.net/search?tn=Nocardioides
