# Supplementary material for: Biocontrol Potential of Endophytic Streptomyces malaysiensis 8ZJF-21 From Medicinal Plant Against Banana Fusarium Wilt Caused by Fusarium oxysporum f. sp. cubense Tropical Race 4
Source: Front Plant Sci. 2022 May 11;13:874819. doi: 10.3389/fpls.2022.874819 (PMC9131080; doi:10.3389/fpls.2022.874819)
Supplement: Supplementary file 1 [file Data_Sheet_1.doc]

# Biocontrol potential of an endophytic *Streptomyces* sp. 8ZJF-21 from medicinal plant against banana Fusarium wilt caused by *Fusarium oxysporum* f. sp. *cubense* tropical race 4

(Lu Zhang, Ziyu Liu, Yong Wang, Jiaqi Zhang, Shujie Wan, Yating Huang, Tianyan Yun, Jianghui Xie, Wei Wang)


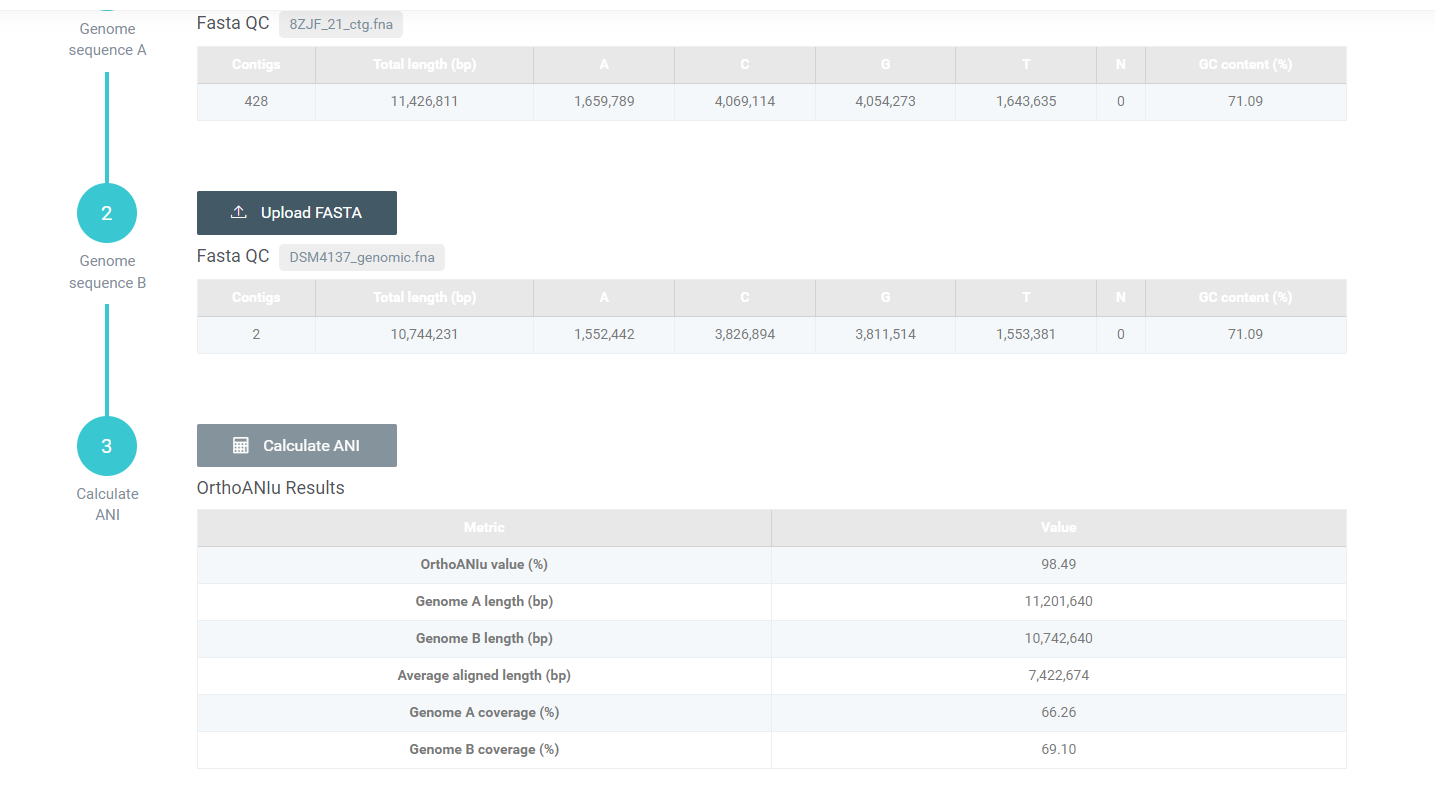


Figure S1 ANI was calculated by comparing the genomes of type strain and stain 8ZJF-21.

**
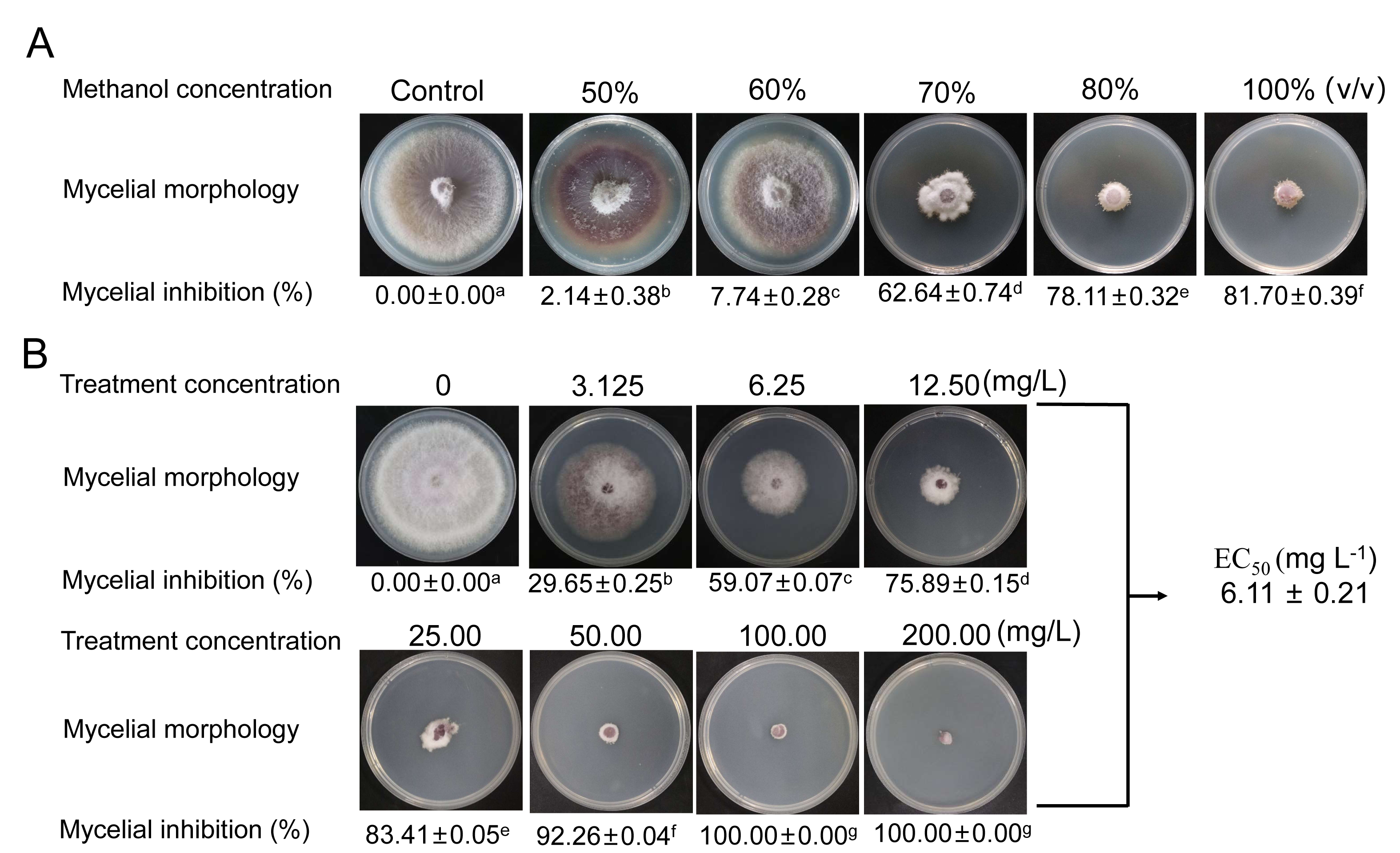
**

Figure S2. Antifungal evaluation of extracts against *Foc* TR4. A, Growth inhibition of *Foc* TR4 by extracts of strain 8ZJF-21 isolated using different gradient methanol solvents. B, Determination of half maximal effective concentration value of extracts against *Foc* TR4.

**
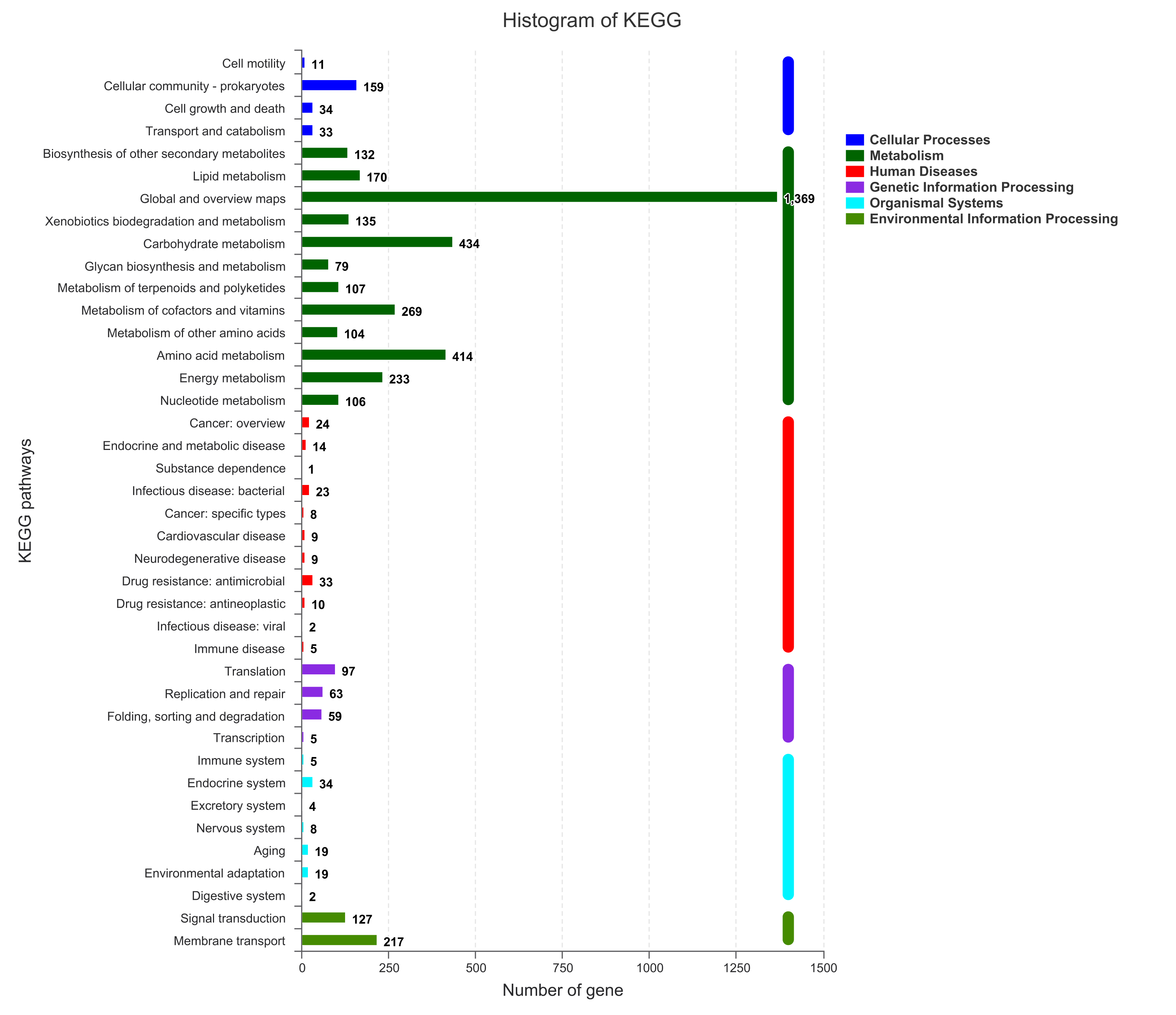
**

Figure S3. KEGG annotation of the strain 8ZJF-21 gnome

**
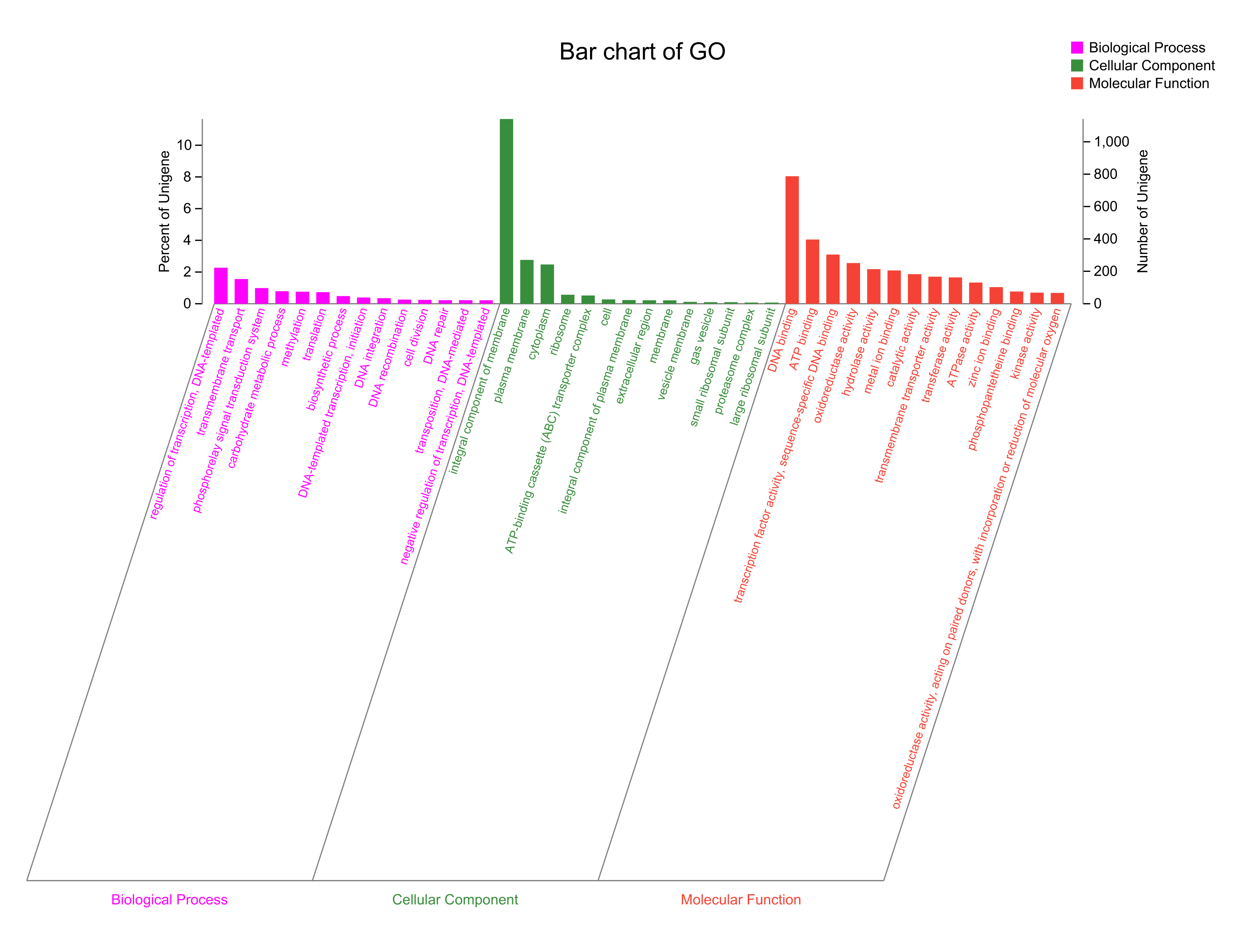
**

Figure S4. GO annotation of the strain 8ZJF-21 gnome

**Table S****1** **Primer sequences used in the study**

| **Gene name** | **Sequence (5’-3’)** | GenBank ID |
| --- | --- | --- |
| *MaMPK1* | GCAAGTGTTCTCCACAGAGAC | XM018826311 |
|  | GTTTCTGAGGTAGTGCGAGCAAGC |  |
| *MaPAL* | GACGTCTCCAGGAACAAGGC | XM009403673 |
|  | CGGAGAACTGCGCGAACATGAGC |  |
| *Maβ-1,3-Glu* | GAGACTCTACGATCCAAACCAAG | AF001523 |
|  | GATTGGAGGCCAGTGACTGC |  |
| *MaPR1* | CTTCACTCCAGCTGCGAAGC | XM009388962 |
|  | GCCTCCTTCGATCGTCTTGTAC |  |
| *MaCAT* | CTACCCGGAGTGGAAGCTCTTC | DQ531612 |
|  | GGCACCACCAGACCCGGGC |  |
| *MaSOD* | GAAGCACCACCAGGCGTAC | JQ411717 |
|  | GTGCACTGTGTGTGGAGGC |  |
| *MaPOD* | GCAGGTATGTCGTGGTTG | EU104681 |
|  | GCATGTCAGGGATCGTACG |  |
| *MaPPO* | GTCACGTCAAGGACGGCG | Mba08_g08740 |
|  | GCGACAGGCTGTGCTTGAAC |  |
| 18S *rRNA* | GTCCCATCCCTTCTGTCGGCGATG | U42083 |
|  | CTGTGATGTTATCCCATGCTAATG |  |

**Table S2** **Growth characteristics of strain 8ZJF-21 on different media**

| Medium | Aerial mycelium | Substrate mycelium | Diffusible pigment | Growth |
| --- | --- | --- | --- | --- |
| ISP2 | Apricot cream | Milky-white | None | ++ |
| ISP3 | Off-white | Greyish white | None | +++ |
| ISP4 | Apricot cream | Milky-white | None | + |
| ISP5 | Milky-white | White | None | ++ |
| ISP6 | Apricot cream | Milky-white | None | +++ |
| ISP7 | Orange yellow | Milky-white | Brown | + |
| PDA | Greyish white | Greyish white | None | ++ |

“+” good; “++” better; “++++” best

**Table S3** **Prediction information of BCGs in strain 8ZJF-21 genome**

| Location | Cluster ID | Type | Start | End | MIBiG accession | Similar Cluster | Similarity (%) | Gene No. |
| --- | --- | --- | --- | --- | --- | --- | --- | --- |
| Scaffold3 | cluster6 | lanthipeptide | 26951 | 101791 | BGC0000093 | meilingmycin | 3 | 62 |
| Scaffold1 | cluster2 | NRPS-like | 328548 | 372715 | BGC0000609 | nocathiacin | 4 | 43 |
| Scaffold91 | cluster48 | T1PKS | 1 | 29483 | BGC0000144 | salinomycin | 6 | 20 |
| Scaffold2 | cluster5 | NRPS | 324029 | 396840 | BGC0000081 | kedarcidin | 7 | 60 |
| Scaffold18 | cluster25 | hserlactone | 1 | 13491 | BGC0001349 | heronamide A-F | 8 | 12 |
| Scaffold7 | cluster16 | butyrolactone | 86770 | 97766 | BGC0001877 | cyphomycin | 9 | 10 |
| Scaffold81 | cluster46 | NRPS | 12675 | 47825 | BGC0001968 | cadaside A/cadaside B | 9 | 27 |
| Scaffold114 | cluster53 | T1PKS | 1 | 20680 | BGC0001215 | conglobatin | 10 | 4 |
| Scaffold1 | cluster1 | caspase | 9413 | 51155 | BGC0000340 | echoside A-E | 11 | 32 |
| Scaffold13 | cluster22 | T1PKS | 66693 | 129157 | BGC0001764 | s56-p1 | 11 | 40 |
| Scaffold93 | cluster50 | T1PKS | 12145 | 38168 | BGC0001183 | lobophorin A | 11 | 12 |
| Scaffold58 | cluster38 | terpene | 9247 | 30309 | BGC0001330 | BE-43547A1-A2/BE-43547B1-B3/BE-43547C1/-2 | 15 | 15 |
| Scaffold1 | cluster3 | nucleoside | 592502 | 650585 | BGC0001065 | herboxidiene | 16 | 37 |
| Scaffold15 | cluster24 | T1PKS | 1 | 61199 | BGC0001447 | primycin | 18 | 47 |
| Scaffold53 | cluster37 | T1PKS | 35631 | 71130 | BGC0000136 | rifamycin | 18 | 22 |
| Scaffold3 | cluster8 | NRPS | 256925 | 335663 | BGC0001792 | surugamide A/surugamide D | 19 | 38 |
| Scaffold7 | cluster17 | T3PKS | 213708 | 258927 | BGC0001807 | totopotensamide A/totopotensamide B | 20 | 30 |
| Scaffold3 | cluster7 | terpene | 210490 | 232218 | BGC0002007 | atolypene A/atolypene B | 23 | 21 |
| Scaffold6 | cluster14 | terpene | 243988 | 273495 | BGC0000065 | rustmicin | 23 | 33 |
| Scaffold7 | cluster15 | NRPS | 1 | 56122 | BGC0000379 | glycinocin A | 23 | 24 |
| Scaffold92 | cluster49 | T1PKS | 1 | 38926 | BGC0000059 | filipin | 23 | 17 |
| Scaffold25 | cluster29 | NRPS-like | 89022 | 119036 | BGC0000074 | herbimycin A | 26 | 20 |
| Scaffold8 | cluster18 | NRPS-like | 1 | 32108 | BGC0000379 | glycinocin A | 27 | 30 |
| Scaffold14 | cluster23 | NRPS | 1 | 40864 | BGC0001011 | meridamycin | 28 | 22 |
| Scaffold42 | cluster34 | T1PKS | 1 | 84812 | BGC0001040 | rapamycin | 28 | 37 |
| Scaffold64 | cluster40 | T1PKS | 1 | 56745 | BGC0000040 | concanamycin A | 28 | 10 |
| Scaffold84 | cluster47 | ladderane | 1 | 36337 | BGC0001975 | atratumycin | 28 | 42 |
| Scaffold101 | cluster51 | T1PKS | 1 | 30605 | BGC0001700 | niphimycins C-E | 29 | 6 |
| Scaffold31 | cluster32 | T1PKS | 1 | 112703 | BGC0000103 | mycolactone B | 33 | 12 |
| Scaffold4 | cluster12 | T1PKS | 279042 | 324951 | BGC0001011 | meridamycin | 42 | 22 |
| Scaffold4 | cluster10 | - | 170 | 60265 | BGC0000028 | bafilomycin B1 | 50 | 21 |
| Scaffold52 | cluster36 | T1PKS | 1 | 49851 | BGC0001662 | mediomycin A | 50 | 20 |
| Scaffold29 | cluster30 | NRPS | 1 | 69066 | BGC0001975 | atratumycin | 55 | 51 |
| Scaffold23 | cluster27 | indole | 12452 | 33601 | BGC0001483 | 5-isoprenylindole-3-carboxylate β-D-glycosyl ester | 61 | 24 |
| Scaffold19 | cluster26 | betalactone | 40729 | 137777 | BGC0000066 | geldanamycin | 73 | 54 |
| Scaffold66 | cluster42 | T2PKS | 1 | 43862 | BGC0000271 | spore pigment | 75 | 44 |
| Scaffold71 | cluster44 | NRPS | 1 | 29807 | BGC0000918 | ochronotic pigment | 75 | 27 |
| Scaffold5 | cluster13 | terpene | 239 | 26765 | BGC0000663 | hopene | 76 | 24 |
| Scaffold30 | cluster31 | T1PKS | 21331 | 101294 | BGC0000053 | elaiophylin | 87 | 37 |
| Scaffold8 | cluster19 | T1PKS | 57546 | 196462 | BGC0001523 | azalomycin F3a | 95 | 47 |
| Scaffold4 | cluster11 | MerR family | 191209 | 271334 | BGC0000075 | hygrocin A/hygrocin B | 96 | 38 |
| Scaffold2 | cluster4 | - | 43744 | 92598 | BGC0000325 | coelichelin | 100 | 37 |
| Scaffold9 | cluster20 | ectoine | 11226 | 21631 | BGC0000853 | ectoine | 100 | 11 |
| Scaffold10 | cluster21 | T1PKS | 34743 | 166903 | BGC0000114 | nigericin | 100 | 52 |
| Scaffold61 | cluster39 | siderophore | 23169 | 34972 | BGC0000941 | desferrioxamin B | 100 | 12 |
| Scaffold65 | cluster41 | NRPS-like | 1 | 35698 | BGC0000340 | echoside A-E | 100 | 30 |
| Scaffold67 | cluster43 | terpene | 1 | 15052 | BGC0001181 | geosmin | 100 | 12 |
| Scaffold78 | cluster45 | terpene | 6264 | 27455 | BGC0001746 | pristinol | 100 | 18 |
| Scaffold24 | cluster28 | terpene | 24848 | 46117 | - | - | - | 19 |
| Scaffold34 | cluster33 | siderophore | 53736 | 65545 | - | - | - | 8 |
| Scaffold47 | cluster35 | siderophore | 46669 | 60498 | - | - | - | 12 |
| Scaffold103 | cluster52 | butyrolactone | 20955 | 29261 | - | - | - | 9 |
